# Image-Guided Stereotactic Radiosurgery for the Treatment of Spasticity and Pain: A Preliminary Experience

**DOI:** 10.7759/cureus.24021

**Published:** 2022-04-11

**Authors:** Pantaleo Romanelli, Giancarlo Beltramo

**Affiliations:** 1 Neurosurgery, Cyberknife Center, Centro Diagnostico Italiano, Milan, ITA; 2 Radiation Oncology, Cyberknife Center, Centro Diagnostico Italiano, Milan, ITA

**Keywords:** brain, trauma, spine, selective dorsal rhizotomy, spinal roots, frameless, image-guidance, stereotactic radiosurgery, spasticity, pain

## Abstract

Background

Spasticity is a major health problem worldwide. Response to current medical and rehabilitation treatments is often poor. Surgical treatment is available only for a very limited number of patients.

Aim

We recently reported the application of stereotactic radiosurgery as a treatment option for spasticity and related pain. This paper describes a larger experience using image-guided stereotactic radiosurgery targeting the cervical or lumbar spinal roots to relieve spasticity and pain in four patients.

Methods

All the patients had refractory spasticity and related pain, one patient had additional paroxystic neuralgic pain. The cause of spasticity and pain was a traumatic brain and/or spinal cord injury, brain and/or spinal cord surgery, and stroke. Symptoms affected the right superior limb in one patient, and the inferior limbs in three patients (unilaterally in two, bilaterally in one). According to the symptoms, one patient was treated at the cervical level (C7 right sensory root) and three patients at lumbar level (right L4, left S1, and L2 roots bilaterally). The target was selected on constructive interference in steady-state (CISS) MR, focusing the irradiation on the postganglionic sensory segment of the cervical root or the intra-foraminal dorsolateral sensory portion of the lumbar roots. Appropriate spasticity and pain scales were used to assess the patient’s status after the treatment.

Results

The treatments were tolerated well. Marked symptomatic relief was found in all the treated patients. Improvements in spasticity and pain scales were observed up to the latest follow-up. After 2 years, the mean reduction of the visual analog scale (VAS) and Modified Ashworth Scale (MAS) was 64.3% and 43.7%, respectively, while the median reduction of MAS score was 50%.

Conclusions

Except for a previous case report, this is the first study describing a novel noninvasive technique based on image-guided radiosurgery to treat severe spasticity and pain due to brain and spinal cord injury. This novel technique appears to be safe and effective and deserves to be studied further.

## Introduction

Stereotactic radiosurgery (SRS) has been historically restricted to the treatment of intracranial targets until the introduction of image-guided frameless radiosurgery by John Adler [1]. The CyberKnife developed by John Adler at Stanford University was the first device to provide frameless SRS with submillimetric accuracy on intracranial and spinal targets [2-4]. Spinal SRS became in shortly a fast-growing field, offering a novel treatment option for patients with spinal tumors, arteriovenous malformations, and metastases [5]. Irradiation of spinal nerve roots to treat functional disorders like spasticity and pain was first reported by our group [6]. This novel radiosurgical technique providing selective dorsolateral irradiation of spinal nerve roots was first used to treat a patient with predominant right leg spasticity following multiple brain and spinal procedures due to recurrent hemangioblastoma. The treatment strategy was developed on the basis of our large experience treating with SRS sensory cranial nerves such as trigeminal or glossopharyngeal nerves to relieve, respectively, trigeminal and glossopharyngeal neuralgia. SRS is a well-established treatment for trigeminal neuralgia (TN) [7]. We have recently published the intermediate and long-term results of image-guided Linac radiosurgery in a large cohort of patients affected by TN [8,9]. On the basis of this experience, we speculated that image-guided radiosurgery could be extended to new targets such as the spinal nerve roots to obtain the radiosurgical equivalent of selective dorsal rhizotomy (SDR).

SDR is a surgical procedure originally developed to treat children with severe spasticity caused by cerebral palsy. SDR has been later applied to the treatment of spasticity in adults, providing symptomatic relief in patients with brain and spinal cord injury [10-13]. During SDR, multiple nerve roots are exposed and neurophysiologically tested. Hyperactive roots undergo selective sensory rhizotomy in order to interrupt the pathologic reflex arc causing spasticity. Image-guided SRS can be similarly used to provide selective lesioning of the sensory component of cervical or lumbar spinal roots. We report here our initial experience treating spasticity and pain due to brain and/or spinal cord injury by stereotactic irradiation of selected spinal roots.

## Materials and methods

Setting and study design

Data were collected and retrospectively analyzed at the CyberKnife Center, Centro Diagnostico Italiano (CDI), Milan.

Participants

The study includes patients undergoing stereotactic spinal root irradiation to relieve spasticity and/or pain.

Radiosurgical treatment

Patients were treated with SRS using a CyberKnife VSI (Accuray Inc., Sunnyvale, CA). High-resolution thin-slice (0.6 mm) computed tomography (CT) was acquired without and with contrast. The treatment planning was carried out on the CT with co-registered constructive interference in steady-state (CISS) and T1-weighted magnetization-prepared rapid gradient echo (MPRAGE) MRI images using MultiPlan (Accuray Inc., Sunnyvale, CA). The treatment planning, including target volume delineation, dose prescription, and optimization of the dose distribution, was executed by an experienced neurosurgeon (PR) and reviewed by a radiation oncologist (GB). An inverse treatment planning algorithm was used to generate steep dose gradients by means of nonisocentric beam delivery with up to 1600 incident beam positions, thereby allowing optimal tumor coverage and minimal dosage to adjacent organs and tissues at risk for late radiation damage.

Target and dose selection

The treating physicians co-registered the CT and MRI datasets and checked the quality of co-registration visually using multiple views and transparency tools of the treatment planning system (TPS) (Multiplan, Accuray Inc.) in the three projections. The selected target (one or more spinal roots) was then identified and drawn. Treatment of cervical roots was performed on the sensory root in the liquoral space between the dorsal ganglion and the root entry zone (postganglionic segment). The target was placed far distal toward the dorsal ganglion to minimize the irradiation of the spinal cord. Treatment of lumbar roots was performed at the intraforaminal level, selecting the dorsolateral sensory portion of the root. Critical volumes, including the spinal cord, thecal sac, kidneys, liver, bowel, and stomach were also delineated to prevent incident beams from passing through them. The prescription dose was 60 Gy in a single fraction for the cervical roots, 35 to 45 Gy for the spinal roots. A lower dose to the lumbar roots was prescribed taking into account the much larger volume of these targets.

Patient evaluation and follow-up

Patient response to treatment was assessed after 3, 6, 12, and 24 months. Spasticity was measured using the Modified Ashworth Scale (MAS), which ranks from 0 (normal muscle tone) to 4 (rigidity). The pain was measured using the visual analog scale (VAS).

## Results

**Figure 1:**
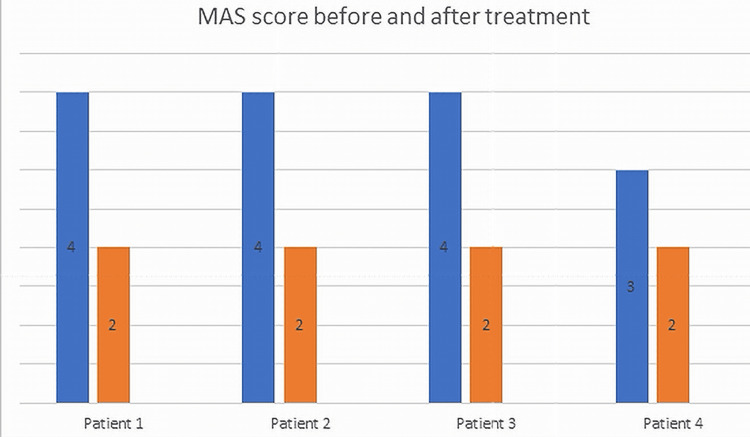
MAS score before and 2 years after the treatment MAS: Modified Ashworth Scale

**Figure 2:**
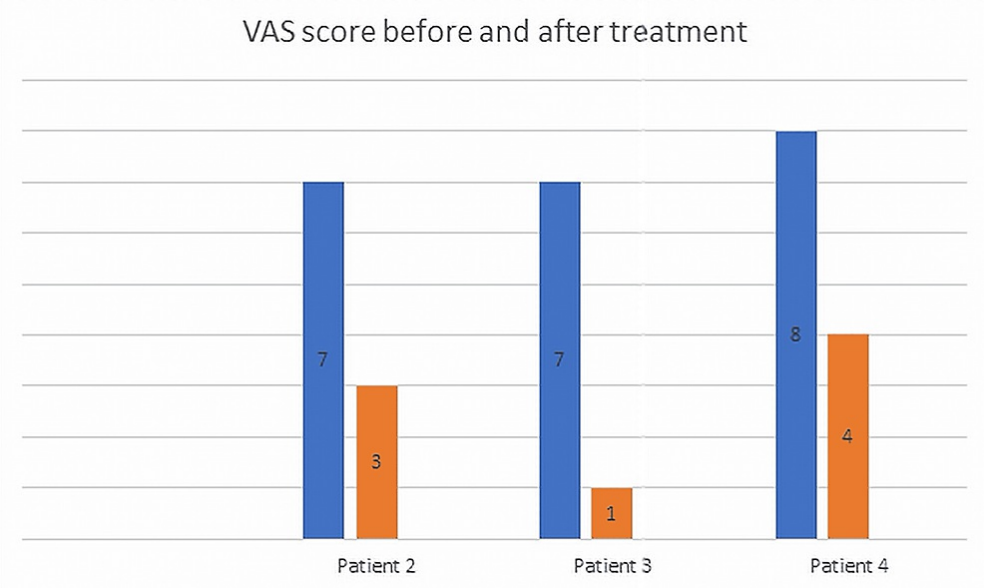
VAS score before and 2 years after treatment VAS: visual analog scale VAS score could be assessed on three patients only, being patient 1 unresponsive.

Four patients affected by spasticity and pain experienced substantial symptomatic relief after SRS targeting the spinal roots. In three patients, there was a marked reduction of spasticity and related pain. In one patient, there was marked reduction of associated neuropathic pain. After 2 years, the mean reduction of VAS and MAS was 64.3% and 43.7%, respectively. The median reduction of MAS score after 2 years was 50%. Treatment involved one cervical root (C6) in one patient and one or more lumbar roots in three patients. Two patients had one lumbar root treated (L4 in one case, S1 in the other) and one patient had two lumbar roots treated (L2 bilaterally). A detailed description of each case is provided below. Figures 1-2 show the MAS and VAS scores, respectively before treatment and after 2 years.

Patient 1

A 34-year-old man had a persistent vegetative state (Glasgow Coma Scale: 8) and spastic quadriplegia after brain injury due to a car accident. He was affected by severe leg flexor spasms, temporarily relieved by lidocaine injection over the L2 roots. His family refused to consider invasive procedures such as surgical rhizotomy or placement of baclofen pumps but accepted SRS. Both L2 roots were treated with 45 Gy delivered to the 75% isodose (mean dose and maximum doses were 52.47 and 60 Gy, respectively, for the right L2 root, 51.93 and 59.91 Gy for the left L2 root). Treatment volume was 409 mm³ for the right and 400 mm³ for the left root. MAS was 4 before treatment, 3 after 3 months, 2 after 6, 12, and 24 months. Caregivers considered these results as a major improvement. VAS could not be assessed, being the patient unresponsive, but the pain was clearly improved due to relief of flexor spasms.

Patient 2

**Figure 3:**
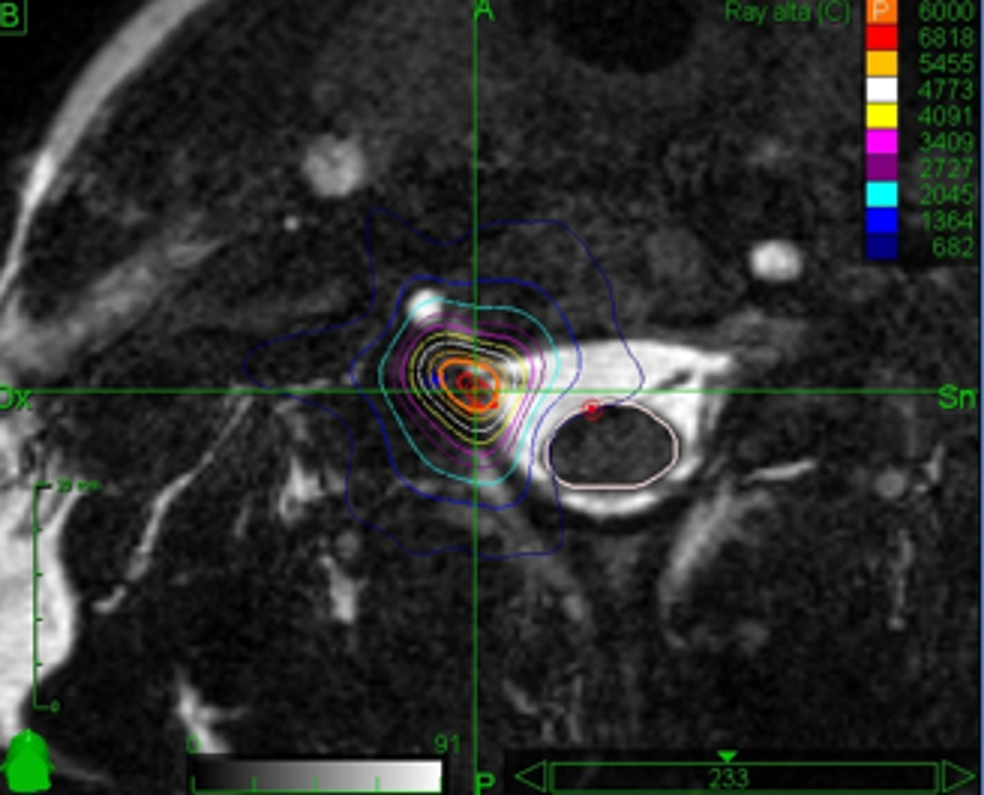
Screenshot of the treatment plan The target (C7 dorsal root) is visible on the axial plane: stereotactic irradiation is focused on the postganglionic segment of sensory root.

A 34-year-old female developed right hemiparesis due to a brain hematoma caused by the bleeding of an arteriovenous malformation. Severe spasticity affecting the right arm and hand could be relieved by botulinum injections over the distribution of the right C7 root but efficacy was lost with time. Due to the refusal of invasive procedures, a selective radiosurgical rhizotomy was proposed and accepted. The right C7 dorsal root was treated with a prescribed dose of 60 Gy, with a mean and maximum dose of 64.2 Gy and 68.18 Gy. The target volume was 57 mm³. The mean and maximum doses delivered to the spinal cord were 1.65 Gy and 7.19 Gy, respectively. Figure 3 shows the treatment plan.

Pre-treatment MAS score was 4. MAS score was 2 after 3 months, 1 after 6, 12, and 24 months. VAS score was 7 before treatment, 2 after 3 months, 1 after 6 months, and 3 after 12 and 24 months.

Patient 3

A 44-year-old man with recurrent hemangioblastomas (von Hippel-Lindau disease) developed spastic tetraparesis following multiple brain and spinal procedures. The right leg was affected by severe spasticity and related pain, not responsive to medical therapy. The patient refused intrathecal baclofen pump. Stereotactic image-guided intraforaminal irradiation was delivered to the dorsolateral portion of the right L4 root, on the basis of electromyography (EMG) findings. The dose delivered was 45 Gy prescribed to the 82% isodose. The mean delivered dose was 50.24 Gy, maximum dose was 54.87 Gy. The target volume was 82 mm³. The treatment was well tolerated, without side effects and complications. Resolution of spasticity and related pain in the right leg was found after 6, 12, 18, and 24 months. Spasticity-related pain disappeared completely within 3 months, going down from 7 to 0 on the VAS score. Spasticity in the right leg was markedly reduced after 6 months, going down from a score of 5 to a score of 1 on the MAS. The MAS score in the contralateral leg improved as well, going down from a score of 4 to a score of 2. VAS and MAS scores remained stable after 12, 18, and 24 months. This case has been extensively described in a detailed case report [6].

Patient 4

**Figure 4:**
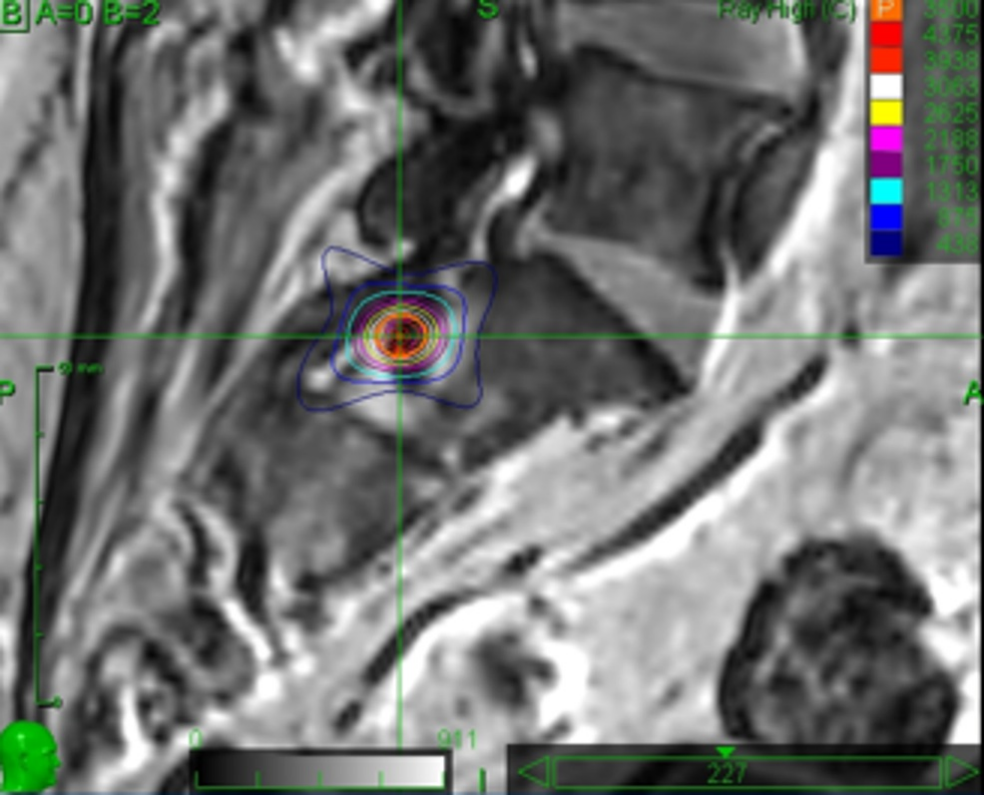
Sagittal view of the treatment planning showing the target (left S1 root)

A 63-year-old male with shooting pain along the left S1 distribution following microsurgical resection of a spinal cord arteriovenous malformation (AVM). VAS score was rated 8. The pain was refractory to medical therapy. He had also spastic paresis of the left leg (MAS score 3). Implant of a spinal cord stimulator was refused. He underwent selective dorsolateral rhizotomy of the left S1 root, receiving 35 Gy prescribed to the 80% isodose. Figure 4 shows a screenshot of the treatment plan.

Mean and maximum doses were 39.42 and 43.75 Gy, respectively. The treatment volume was 105 mm³. VAS score went down to 3 after 3 months, 2 after 6 and 12 months, and 4 after 24 months. MAS score went down to 2 after 3 months and remained stable thereafter.

## Discussion

Close to 100 million people worldwide suffer the sequelae of severe trauma to the brain or spinal cord, with spasticity and related pain being a common long-term complication in survivors [14]. This paper reports on a novel noninvasive treatment for spasticity: image-guided SRS of spinal nerve roots. This new treatment has been developed to offer the radiosurgical equivalent of SDR, an established surgical procedure to treat spasticity in children and adults affected by brain and/or spinal cord injury [11-14]. Spasticity is caused by the failure of upper motor neurons to control the discharge of lower motor neurons. The increased firing by the lower motor neurons following the loss of supraspinal control operated by the corticospinal, reticulospinal, and vestibulospinal tracts is sustained by the hyperactivity of the reflex arc with consequent increase of muscle tone (hypertonus) and spinal stretch reflex (hyperreflexia), clonus, and painful muscle spasms in response to stretch and cutaneous stimulation.

SDR counteracts spasticity by removing the afferent inputs of the reflex arc, brought to the spinal motor neurons by the sensory fibers (Ia) located in the dorsolateral portion of the spinal roots. SDR is a relatively invasive procedure requiring multiple laminectomies to expose the dural sac, which is opened to allow the exposure and section of the involved rootlets. The selection of the rootlets to be sacrificed is guided by intraoperative electrophysiological mapping. Preoperative EMG and botulinum injections are useful to identify the dorsal roots involved in the genesis of spasticity and to predict the clinical efficacy of the procedure. An alternative to SDR, especially in adult patients, is the placement of an intrathecal baclofen pump. The main shortcoming of this procedure is the need to implant hardware and to periodically refill the reservoir: missing baclofen refill appointments can lead to severe and potentially fatal withdrawal syndrome. Also, hardware-related complications are relatively common. The cost and maintenance of the baclofen pump can be an issue in several regions of the world, essentially restricting its use in wealthy countries.

Image-guided frameless SRS is a precise, noninvasive, and highly cost-effective procedure and is an emerging treatment for functional central nervous system (CNS) disorders (such as pain and epilepsy). The role of SRS in the treatment of TN is well established [7]. CyberKnife® (Accuray Incorporated, Sunnyvale, California) frameless image-guided robotic radiosurgery is currently the less invasive non-medical treatment for TN, providing robust and durable pain control and absence of major neurological complications [8-9]. CyberKnife® radiosurgery can be used safely in any body region, allowing the precise and accurate treatment of targets unreachable by other radiosurgical platforms. Cervical or lumbar sensory roots can be treated by CyberKnife® radiosurgery as safely as any intracranial target.

Image-guided SRS of selected nerve roots to relieve spasticity and pain is an interesting noninvasive alternative to SDR or to the placement of a baclofen pump. The treatment delivered doses ranging from 35 to 45 Gy to the dorsolateral portion of the lumbar spinal roots, characterized by a relatively large target volume, while the same dose was used to treat the trigeminal nerve (60 Gy) was used to treat the sensory cervical root. Both doses are not strictly ablative and the clinical benefit is likely to be mediated by the non-necrotic effects of radiation. Radiomodulation has been suggested to be related to symptomatic relief in patients undergoing SRS for epilepsy and TN [14]. It’s indeed intriguing to speculate that the clinical improvements after spinal roots SRS are possibly mediated by a radiomodulation process.

Image-guided SRS of the sensory component of spinal nerve roots appears to be a safe procedure due to the small target volume involved, with minimal irradiation of adjacent organs. However, a precise assessment of toxicity related to the treatment requires a prospective study on a much larger cohort of patients. On the basis of the experience treating TN with SRS, the development of hypoesthesia and/or paresthesia can be expected in some patients.

The preliminary experience here discussed has shown that SRS of the spinal nerves provided benefits in patients with brain and/or spinal cord injury due to a variety of causes, including stroke, trauma, and surgery. Further work is needed to assess the indications of this technique and define which conditions may benefit most from this treatment. This study is limited by the small number of patients and the relatively heterogeneous origin of spasticity. Further studies are being planned to understand further the indications, benefits, and limits of this novel technique.

## Conclusions

Stereotactic image-guided SRS of cervical and spinal nerve roots appears to be a safe and effective noninvasive treatment for patients with spasticity and pain caused by brain or spinal cord injury. This technique provides a useful option for the treatment of a wide variety of patients suffering from the long-term sequelae of neurological injury. Further studies to evaluate this novel treatment option are needed.

## References

[1] Romanelli P, Schaal DW, Adler JR (2006). Image-guided radiosurgical ablation of intra- and extra-cranial lesions. Technol Cancer Res Treat.

[2] Chang SD, Main W, Martin DP, Gibbs IC, Heilbrun MP (2003). An analysis of the accuracy of the CyberKnife: a robotic frameless stereotactic radiosurgical system. Neurosurgery.

[3] Romanelli P, Schweikard A, Schlaefer A, Adler J (2006). Computer aided robotic radiosurgery. Comput Aided Surg.

[4] Fürweger C, Drexler C, Kufeld M, Muacevic A, Wowra B, Schlaefer A (2010). Patient motion and targeting accuracy in robotic spinal radiosurgery: 260 single-fraction fiducial-free cases. Int J Radiat Oncol Biol Phys.

[5] Romanelli P, Adler JR Jr (2008). Technology Insight: image-guided robotic radiosurgery--a new approach for noninvasive ablation of spinal lesions. Nat Clin Pract Oncol.

[6] Romanelli P, Beltramo G (2020). Stereotactic dorsolateral irradiation of spinal nerve roots: a novel technique for the treatment of spasticity and pain. Cureus.

[7] Tuleasca C, Régis J, Sahgal A (2018). Stereotactic radiosurgery for trigeminal neuralgia: a systematic review. J Neurosurg.

[8] Romanelli P, Conti A, Redaelli I, Martinotti AS, Bergantin A, Bianchi LC, Beltramo G (2019). Cyberknife radiosurgery for trigeminal neuralgia. Cureus.

[9] Romanelli P, Conti A, Bianchi L, Bergantin A, Martinotti A, Beltramo G (2018). Image-guided robotic radiosurgery for trigeminal neuralgia. Neurosurgery.

[10] Gump WC, Mutchnick IS, Moriarty TM (2013). Selective dorsal rhizotomy for spasticity not associated with cerebral palsy: reconsideration of surgical inclusion criteria. Neurosurg Focus.

[11] Park TS, Dobbs MB, Cho J (2018). Evidence supporting selective dorsal rhizotomy for treatment of spastic cerebral palsy. Cureus.

[12] Enslin JM, Langerak NG, Fieggen AG (2019). The evolution of selective dorsal rhizotomy for the management of spasticity. Neurotherapeutics.

[13] Agrawal M, Samala R, Doddamani R, Agrawal D, Chandra SP (2021). The role of selective dorsal rhizotomy in the management of post-traumatic spasticity: systematic review. Neurosurg Rev.

[14] Régis J, Carron R, Park M (2010). Is radiosurgery a neuromodulation therapy? : A 2009 Fabrikant award lecture. J Neurooncol.

